# Hypoglycaemia and neonatal brain injury

**DOI:** 10.1186/1824-7288-40-S2-A13

**Published:** 2014-10-09

**Authors:** James P Boardman

**Affiliations:** 1 MRC Centre for Reproductive Health, University of Edinburgh, Edinburgh EH16 4TJ, UK

## 

The transition from fetal to neonatal life requires metabolic adaptation to ensure energy supply to vital organs and systems after separation from the placental circulation. Under normal conditions this is achieved through the mobilisation and use of alternative cerebral fuels (fatty acids, ketone bodies and lactate) when blood glucose concentration falls.

The level of blood glucose (BG) concentration that leads to cerebral injury in newborns and adverse neurodevelopmental outcome is unknown: low BG is often observed during postnatal adaptation of healthy term infants without apparent adverse consequence, and the capacity to mobilise and use alternative cerebral fuels when BG is low varies between patient groups. Severe hypoxic-ischaemic encephalopathy (HIE) is associated with impaired metabolic adaptation, and animal and human data suggest that levels of hypoglycaemia that are tolerated under normal conditions may be harmful in association with hypoxia-ischaemia.

The spectrum of cerebral injury associated with hypoglycaemia is wide and includes: white matter injury including parenchymal haemorrhage and ischaemic stroke, cortical neuronal injury, and sometimes signal change in the basal ganglia (mainly the globus pallidus) and thalami. Vulnerability of the white matter and cortex of the posterior parietal and occipital lobes has been well reported in human imaging studies, but the site of injury is more widespread in pathological and experimental studies of neonatal hypoglycaemia. In the largest series of infants with isolated neonatal hypoglycaemia and acute neurological dysfunction, there was an association with a predominantly posterior pattern of injury in one third of the cohort, and a more extensive distribution of lesions was common.

Safe clinical management relies on the identification of infants at risk of neurological sequelae from hypoglycaemia, adequate energy provision after birth, monitoring of blood glucose, and prompt intervention to raise the BG at specified thresholds, with the caveat that acute neurological dysfunction in association with low BG at any level should prompt urgent investigation and treatment. The optimal target blood glucose level for ensuring adequate energy provision in health and in HIE remains unknown. However, recent data support guidance to maintain blood glucose concentration ≥2.5mmol/L in neonates with signs of acute neurological dysfunction, which includes those with HIE, and is higher than the accepted threshold of ≥2mmol/L in infants without abnormal signs or hyperinsulinism.

